# Chromatin 3D structure, phase separation and disease

**DOI:** 10.1093/lifemedi/lnad010

**Published:** 2023-03-08

**Authors:** Lili Fan, Xinyi Liu, Diana Guallar, Junjun Ding

**Affiliations:** Guangzhou Key Laboratory of Formula-Pattern of Traditional Chinese Medicine, School of Traditional Chinese Medicine, Jinan University, Guangzhou 510632, China; RNA Biomedical Institute, Sun Yat-sen Memorial Hospital, Zhongshan School of Medicine, Sun Yat-sen University, Guangzhou 510080, China; Advanced Medical Technology Center, The First Affiliated Hospital, Zhongshan School of Medicine, Sun Yat-sen University, Guangzhou 510006, China; Center for Stem Cell Biology and Tissue Engineering, Key Laboratory for Stem Cells and Tissue Engineering, Ministry of Education, Zhongshan School of Medicine, Sun Yat-sen University, Guangzhou 510006, China; Center for Research in Molecular Medicine and Chronic Diseases, Universidade de Santiago de Compostela, Santiago de Compostela 15782, Spain; RNA Biomedical Institute, Sun Yat-sen Memorial Hospital, Zhongshan School of Medicine, Sun Yat-sen University, Guangzhou 510080, China; Advanced Medical Technology Center, The First Affiliated Hospital, Zhongshan School of Medicine, Sun Yat-sen University, Guangzhou 510006, China; Center for Stem Cell Biology and Tissue Engineering, Key Laboratory for Stem Cells and Tissue Engineering, Ministry of Education, Zhongshan School of Medicine, Sun Yat-sen University, Guangzhou 510006, China; Department of Histology and Embryology, School of Basic Medical Sciences, Guangzhou Medical University, Guangzhou 511436, China; West China Biomedical Big Data Center, West China Hospital, Sichuan University, Chengdu 610041, China

In recent years, several studies have described dynamic changes in chromatin 3D structure and intracellular phase separation during disease development. These changes are closely related to alterations in gene expression and therefore are proposed as one of the molecular basis of disease occurrence and cell fate determination. Chromatin is spatially organized into three-dimensional structures at different levels, such as A/B compartments, topologically associating domains (TADs) and chromatin loops. Liquid–liquid phase separation (LLPS) is a physical concept that two distinct phases are formed from homogeneous mixture, scientists have discovered that it also appears inside the cell. In cells, proteins and other biological macromolecules of similar properties form droplets condensate in the cytoplasm and nucleus. Dysregulation of LLPS and chromatin 3D structure can lead to many diseases, especially cancer, developmental disorders, and heart diseases. Further, an increasing number of studies have revealed that LLPS regulates chromatin 3D structure, which may be one of the molecular mechanism of diseases. Thus, is it crucial to understand how LLPS regulates chromatin 3D structure in order to explore the mechanisms underlying disease progression. To this end, we summarized the dynamics of chromatin 3D structure and LLPS in diseases and proposed possible models explaining the mechanistic basis.

Disease occurrence and development are abnormal cell fate transition processes. Recent studies have elucidated the role of chromatin 3D structure and LLPS during normal cell fate transition, which may provide some insights into disease development. Somatic cells can be reprogrammed into induced pluripotent stem cells (iPSC) after transducing pluripotency-related transcription factors or other methods. One study showed that LLPS of the transcription factor OCT4 regulates TAD reorganization to induce somatic cell reprogramming [4]. Accordingly, the efficiency of somatic cell reprogramming can be improved by manipulating OCT4 phase separation or TAD fusion. In addition, another study showed that LLPS of the structural regulator CTCF mediates chromatin interactions between A compartments to regulate other cell fate transition processes, such as the self-renewal of mouse embryonic stem cells and differentiation toward neural progenitor cells [5]. In brief, the chromatin 3D structure and the phase separation play some roles during cell fate transitions.

Aberrant chromatin 3D structure has been linked to many diseases, especially developmental disorders, premature aging, and cancer [2].

Developmental disorders can be caused by the disruption of chromatin 3D structure, which alters the expression of developmental stage-specific genes and leads to aberrant cell fate transition. For example, congenital limb deformities can be caused by mutations in the *ZRS* enhancer or deletion of the surrounding CTCF binding sites. Both events can lead to reduced interaction between *SHH* gene of sonic hedgehog signaling molecule and *ZRS* enhancer, eventually leading to dysregulated *SHH* expression and congenital limb deformities.

In addition to developmental disorders, disruption of chromatin 3D structure can also lead to cellular senescence through altered gene expression, ultimately leading to a series of related diseases such as progeria. Progeria laminopathy is caused by a point mutation at *LMNA* gene, which results in an alternatively spliced isoform called Progerin that produces nuclear instability and premature aging. In particular, the intensity of chromatin compartmentalization and the interaction of Lamin A/C with heterochromatin are lowered globally compared to normal cell lines.

Furthermore, aberrant chromatin 3D structure can lead to carcinogenesis at different hierarchical structures. For example, nearly 12% of genomic regions in breast cancer cells show compartment switching. Deletion of the TAD boundary results in activation of the *TAL1* and *LMO2*, ultimately leading to T-cell acute lymphoblastic leukemia. Moreover, aberrant binding of structural factors on the genome leads to the disassembly of TADs and chromatin loops, ultimately leading to cancer. Hypermethylation of the structural factor CTCF binding sites results in loss of CTCF binding at TAD boundaries, which leads to aberrant activation of the oncogene *PDGFRA* through its persistent interaction with its enhancer, which ultimately induces gliomagenesis. Those examples show that in some cases aberrant chromatin 3D structures can lead to disease, and more investigation of in-depth mechanisms in other cases is still needed.

**Figure 1.**
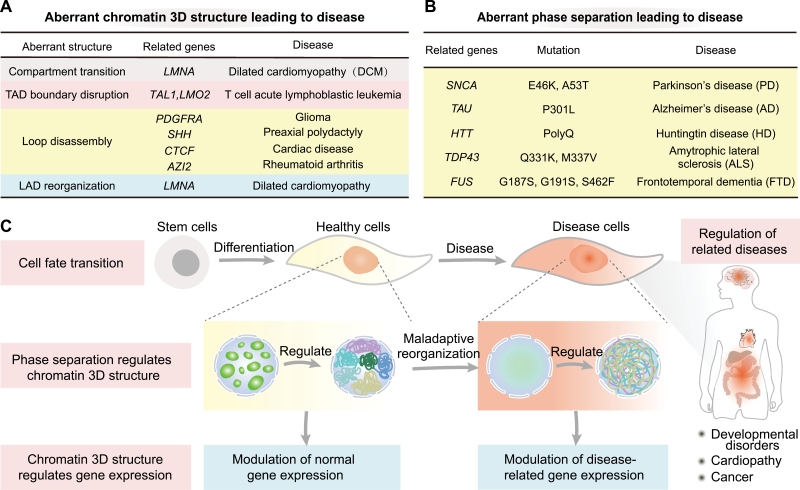
**Phase separation is associate with the disease through chromatin 3D structure.** (A) Table of aberrant chromatin 3D structure leading to disease. (B) Table of aberrant phase separation leading to disease. (C) A possible model that phase separation regulates gene expression through chromatin 3D structure in physiological conditions, thereby controlling cell fate transition, and aberrations in this process can lead to disease.

Finally, there are correlations between aberrant chromatin 3D structure and heart, endocrine, autoimmune, and infectious diseases, as well as laminopathies. Another mutation in the *LMNA* gene in cardiac patients enhances A/B compartment separation and alters the ratio of Lamin-associated domains (LADs). The redistributed LADs increase CpG methylation levels to suppress gene expression, ultimately inducing dilated cardiomyopathy. Heart failure is severe heart disease with dramatic chromatin 3D structure, which is closely related to the downregulation of CTCF in patients (Fig. 1A).

Aberrant phase separation and the ensuing aggregation of proteins have been linked to neurodegenerative diseases, cancer, and other diseases [1]. Indeed, Insoluble aggregation of proteins is a typical pathological feature of neurodegenerative disorders, such as alpha-synuclein (SNCA) aggregates in Parkinson’s disease, TAU aggregates in Alzheimer’s disease, huntingtin (HTT) aggregation in Huntington’s disease, and granular protein aggregation in stress amyotrophic lateral sclerosis and frontotemporal dementia (FTD). On the other hand, changes in the position, composition, or aggregation state of phase separation caused by genetic mutations can drive cancer development. Several studies have shown that histone mutation H3K27M caused brainstem gliomas, and abnormal destruction of its chromatin LLPS condensates is one of the mechanisms. Therefore, understanding the mechanism of protein phase separation in diseases can identify new therapeutic targets (Fig. 1B).

Recent studies have shown that intranuclear phase separation can directly regulate the chromatin 3D structure. In particular, our study demonstrated that the phase separation ability of the pluripotent factor OCT4 contributes to somatic reprogramming by regulating TAD reorganization [4], and the structural factor CTCF can regulate long-range chromatin interactions between A compartments through phase separation [5]. Disrupting global phase separation in a cell can also alter the chromatin structure at compartment, TAD, and long-range interaction levels [3]. In diseases, several studies have shown that NUP98 and FUS undergo LLPS on super-enhancers related to oncogenes, and thus drive carcinogenesis. Based on these findings, we hypothesized that phase separation regulates gene expression through chromatin 3D structure in physiological conditions, thereby controlling cell fate transition, and aberrations in this process can lead to disease (Fig. 1C).

At present, whether phase separation regulates chromatin 3D structure has attracted more and more attention, especially because it remains to be determined why phase separation is one of the mechanisms of chromatin regulator functions. On the other hand, further studies are needed to elucidate the role of chromatin 3D structure in the regulation of phase separation, and new methods and algorithms must be developed to further resolve the regulatory relationship between the two. Most importantly, the mechanisms of how phase separation and chromatin 3D structure cooperate to cause disease is unclear and deserves further study.
